# Patterns of incident Burkitt lymphoma during the HIV epidemic among the Black African and White population in South Africa

**DOI:** 10.1038/s41416-024-02937-8

**Published:** 2025-01-14

**Authors:** Carole Metekoua, Yann Ruffieux, Judith Mwansa-Kambafwile, Patricia Kellett, Matthias Egger, Mazvita Muchengeti, Eliane Rohner, Tracey Wiggill

**Affiliations:** 1 National Cancer Registry, National Health Laboratory Service, Johannesburg, South Africa; 2 Graduate School for Health Sciences, University of Bern, Bern, Switzerland; 3 Institute of Social and Preventive Medicine (ISPM), University of Bern, Bern, Switzerland; 4 School of Public Health, University of the Witwatersrand, Johannesburg, South Africa; 5 Population Health Sciences, Bristol Medical School, University of Bristol, Bristol, UK; 6 Centre for Infectious Disease Epidemiology and Research (CIDER), School of Public Health and Family Medicine, University of Cape Town, Cape Town, South Africa; 7 South African DSI-NRF Centre of Excellence in Epidemiological Modelling and Analysis (SACEMA), Stellenbosch University, Stellenbosch, South Africa; 8 Medical Microbiology and Immunology, National Health Laboratory Service, Tygerberg Hospital, Cape Town, South Africa; 9 Faculty of Medicine and Health Sciences, Stellenbosch University, Stellenbosch, South Africa

**Keywords:** Epidemiology, B-cell lymphoma

## Abstract

**Background:**

Burkitt lymphoma (BL) may be HIV-associated but data on BL trends in South Africa (SA), where HIV is highly prevalent, are scarce. We compared BL incidence trends over 36 years among Black African and White individuals.

**Methods:**

We included histologically diagnosed BL from the National Cancer Registry in SA between 1986–2021. We computed yearly age-standardised incidence rates (ASIR) by race, and annual percentage changes in ASIR using Joinpoint regression.

**Results:**

Between 1986–2021, 2205 Black African (ASIR: 1.68/1,000,000; 95% confidence interval [CI] 1.63–1.73) and 366 White individuals (ASIR: 2.34/1,000,000; 95% CI 2.15–2.53) had incident BL. Median age at diagnosis increased over time, while the male proportion among those diagnosed declined. The ASIR among Black Africans increased from 1986–2012 and declined thereafter with BL incidence peaks shifting from children and elderly to middle-aged adults. Among White individuals, BL rates rose among all age groups over time.

**Conclusions:**

The BL epidemiology among Black Africans, with decreasing rates since 2012, may reflect SA’s evolving HIV epidemic. In contrast, BL rates among White individuals in SA and many high-income countries continue to increase over time. Further studies are needed to better understand the differences in BL epidemiology across geographic regions and population groups.

## Background

Burkitt lymphoma (BL) is a highly aggressive type of B-cell non-Hodgkin lymphoma [1]. Risk factors for BL include infection with Epstein Barr virus (EBV) or the malaria-causing parasite *Plasmodium falciparum*, and immunodeficiency [2]. In most countries, BL shows a male predominance with two- to four-fold higher incidence rates in male than female individuals. The age-specific BL incidence rates usually peak among children and the elderly and in certain populations an additional peak in adulthood is observed [3]. Geographically, BL rates exhibit a mosaic pattern [3, 4]. Africa and Europe include countries with high age-standardised incidence rate [ASIR] (>4 cases per million people), intermediate (2–3.9 cases per million people), and low (<2 cases per million people) BL rates while in Asia, North America, South America and Central America, BL rates are typically low or intermediate [3]. Mortality associated with BL is higher in low- and middle-income countries compared to high-income countries [4, 5], probably due to late BL diagnosis and less effective treatment [4].

People with HIV (PWH) are at a higher risk of developing BL than the general population [6, 7]. HIV can increase the risk of BL through different mechanisms. The activities of HIV-1 in infected cells produce proteins that are transported to uninfected B-cells where they promote gene instability, which may be associated with the acquisition of a translocation involving the *c-MYC* proto-oncogene and the immunoglobulin heavy or light chain gene loci [8, 9]. HIV also promotes the proliferation of EBV-infected cells [10]. BL incidence rates were high among PWH in the United States (US) while the HIV epidemic was uncontrolled in the 1980’s. At that time, BL was characterised as an AIDS-defining malignancy [11, 12]. When anti-retroviral therapy (ART) was introduced, the BL rates among PWH in the US stabilised [11, 13]. Yet, the changes in the epidemiology of HIV-related BL in the US may not apply to sub-Saharan Africa, given that the aetiology of BL varies across these regions. In sub-Saharan Africa, HIV-related BL is more frequently associated with EBV than in high-income countries [14]. Furthermore, South Africa has the highest number of PWH globally with approximately 7.9 million individuals living with HIV in 2017 [15]. In that year, 16.6% of Black African but only 1.1% of White individuals in South Africa lived with HIV [15]. In South Africa, up to 95% of patients diagnosed with BL live with HIV [16, 17] compared to 23% in the US [18].

Although the vast majority of PWH live in sub-Saharan Africa, few studies have assessed the temporal trend of BL incidence rates in this region. We used data from South Africa’s National Cancer Registry (NCR) to examine trends of BL incidence rates over 36 years in relation to the evolving HIV epidemic among Black African and White individuals.

## Methods

### Data source

We used data from the NCR to identify BL diagnoses among Black African and White individuals in South Africa between 1986–2021. The NCR has been described in detail elsewhere [19]. Briefly, the NCR was established in 1986, and by 2011, the National Health Act of South Africa declared cancer to be a notifiable disease and mandated all cancers be reported to the NCR. For this study, we used data from the nation-wide pathology registry, which receives complete laboratory reports of cancers diagnosed in public and private laboratories by histopathology, cytopathology, bone marrow aspirate, or trephine. Coders at the NCR rely on the International Classification of Diseases for Oncology, 3rd edition (ICD-O-3) to classify these reports [20]. The NCR uses race as captured in the laboratory forms and hot-deck imputation to assign race where this information is missing [20, 21].

We obtained annual mid-year population data for the Black and White population from the NCR archives (1986–2001) and from Statistics South Africa (Stats SA) (2002–2021), the national statistics agency of South Africa [22].

### Inclusion criteria and definitions

We identified BL diagnoses assigned ICD-O-3 morphology codes 9687 (Burkitt lymphoma, not otherwise specified) or 9826 (Burkitt cell leukaemia) from Black African and White individuals of any age. We excluded records without information on race and records from Indian/Asians and individuals of mixed race due to the limited number of records available.

We divided the study population into 16 age categories (0–4, 5–9, 10–14,…,≥75 years) and five broad life cycle stages: children (0–14 years), youth (15–24 years), young adults (25–44 years), middle age (45–64 years), and elderly (≥65 years). We defined four calendar periods based on HIV prevalence estimates from the Thembisa model [23] and the main eras of pre-ART and ART in South Africa. The pre-ART era comprised 1986–1991 with low HIV prevalence (<1%) and 1992–2003 with increasing HIV prevalence from 1% to 9%. The ART era comprised 2004–2015 with free ART in public health facilities based on predefined criteria, and 2016–2021, characterised by the Universal Test and Treat (UTT) policy [24].

### Statistical analysis

We used descriptive statistics to examine BL characteristics across calendar periods. We computed age-specific BL incidence rates with the 16 age groups, stratified by calendar period and race. We computed yearly ASIR stratified by race using direct standardisation with the mid-year population being the denominator and the Segi’s world standard population of 1960 [25] being the population of reference. We aggregated the weights in the 75–79, 80–84, and ≥85 age groups in Segi’s population to fit the age group distribution in our dataset. In 1987, the NCR recorded an abnormally low number of non-Hodgkin lymphoma diagnoses, suggesting a problem with that year’s data capture. Therefore, we used data from 1986 and 1988 to interpolate the number of BL and the mid-year population for 1987. We used the ASIR as input data for Joinpoint Trend Analysis Software Version 5.0.2, which allowed us to compute the annual percentage change (APC) and identify ASIR trends over time. To identify the adequate number of joinpoints, we used the default software settings. We computed age-adjusted incidence rate ratios (IRR) comparing the BL rates in male and female individuals over the defined calendar periods. We plotted key results against the changes in HIV prevalence and ART coverage in the general population based on the Thembisa model version 4.6 [23].

## Results

Between 1986–2021, 3026 individuals were newly diagnosed with BL in South Africa. Of those, we excluded 278 (9%) individuals of mixed race, 46 (2%) Indian/Asians, and 131 (4%) individuals with missing information on race. We included 2205 Black African and 366 White individuals with an incident BL diagnosis for a total of 2571 individuals in our analysis.

**Fig. 1:**
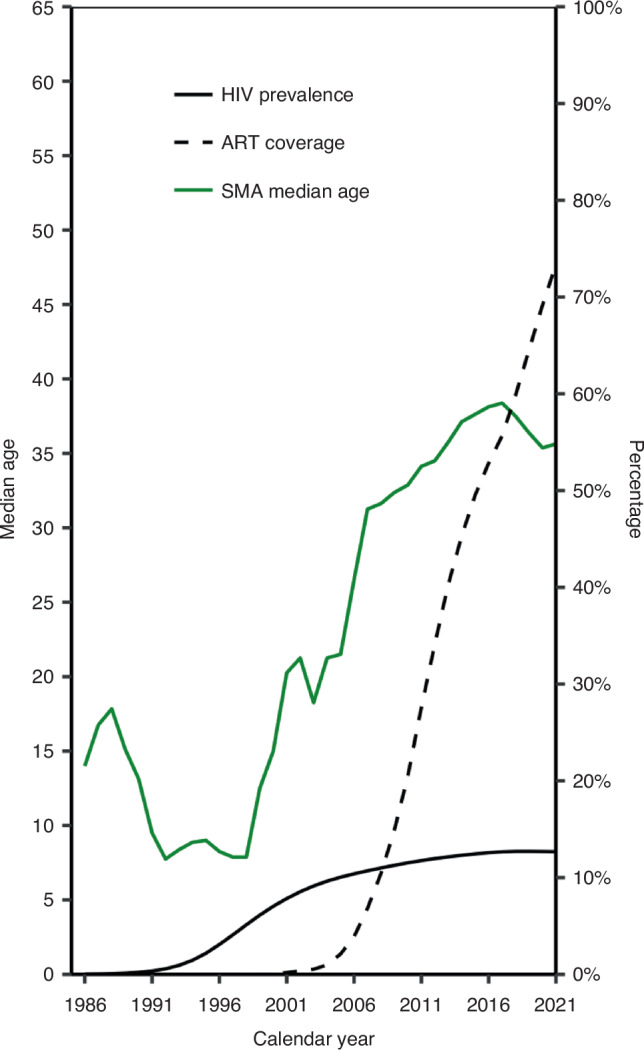
Changes in median age at Burkitt lymphoma diagnosis over calendar years. SMA simple moving average over 5 years periods, ART anti-retroviral treatment, HIV human immunodeficiency virus.

Patient characteristics varied across calendar periods. The median age at BL diagnosis increased between 1986–2016 and slightly declined thereafter (Fig. 1). Median age at BL diagnosis was 7 years (interquartile range [IQR] = 5–21) in 1986–1991, rising to >30 years in 2004–2015 and 2016–2021 (Table 1). Two-thirds of those diagnosed with BL in 1986–1991 (*n* = 48; 66.7%) were children, but only one in six diagnosed with BL between 2004–2015 were children. In the two most recent calendar periods, over half of all BL diagnoses occurred in young adults (24–44 years). In 1986–1991, most BL diagnoses occurred in male individuals (67.4%; *n* = 60), whereas between 2004–2015, approximately half of all BL diagnoses occurred in female individuals (*n* = 730; 49.6%). The proportion of Black Africans among individuals diagnosed with BL increased from 70.2% (*n* = 66) in 1986–1991 to 89.7% (*n* = 1321) in 2004–2015 and decreased to 84.9% (*n* = 527) thereafter.

**Table 1:** Characteristics of study population by calendar period.

Characteristics	1986–1991	1992–2003	2004–2015	2016–2021	Total
	(*N* = 94)	(*N* = 382)	(*N* = 1473)	(*N* = 622)	(*N* = 2571)
Median age (IQR) [years]	7 (5, 21)	12 (5, 33)	35 (25, 43)	36 (26, 44)	33 (16, 42)
Age at BL diagnosis [years]					
Children (0–14)	48 (66.7%)	200 (53.6%)	241 (16.6%)	107 (17.3%)	596 (23.7%)
Youths (15–24)	9 (12.5%)	34 (9.1%)	104 (7.2%)	38 (6.1%)	185 (7.4%)
Young adult (25–44)	5 (6.9%)	101 (27.1%)	793 (54.6%)	326 (52.7%)	1225 (48.7%)
Middle age (45–64)	6 (8.3%)	33 (8.8%)	288 (19.8%)	133 (21.5%)	460 (18.3%)
Elderly (65+)	4 (5.6%)	5 (1.3%)	26 (1.8%)	15 (2.4%)	50 (2.0%)
Missing^a^	22	9	21	3	55
Sex					
Female	29 (32.6%)	146 (39.9%)	730 (49.6%)	281 (45.2%)	1186 (46.5%)
Male	60 (67.4%)	220 (60.1%)	742 (50.4%)	341 (54.8%)	1363 (53.5%)
Missing^a^	5	16	1	0	22
Race/population group					
Black African	66 (70.2%)	291 (76.2%)	1321 (89.7%)	527 (84.7%)	2205 (85.8%)
White	28 (29.8%)	91 (23.8%)	152 (10.3%)	95 (15.3%)	366 (14.2%)
Burkitt lymphoma site					
Nodal	**94 (100.0%)**	**274 (73.9%)**	**826 (56.3%)**	**466 (75.4%)**	**1660 (65.1%)**
Head, face and neck	0	17 (40.5%)	157 (36.9%)	89 (31.0%)	263 (34.8%)
Abdomen	0	7 (16.7%)	72 (16.9%)	58 (20.2%)	137 (18.1%)
Thorax	0	1 (2.4%)	28 (6.6%)	8 (2.8%)	37 (4.9%)
Pelvic	0	0 (0.0%)	9 (2.1%)	9 (3.1%)	18 (2.4%)
Axilla and arm	0	10 (23.8%)	124 (29.1%)	83 (28.9%)	217 (28.7%)
Inguinal region or leg	0	5 (11.9%)	36 (8.5%)	40 (13.9%)	81 (10.7%)
Multiple sites	0	2 (4.8%)	0 (0.0%)	0 (0.0%)	2 (0.3%)
NOS	94	232	400	179	905
Extranodal	**0 (0.0%)**	**97 (26.1%)**	**640 (43.7%)**	**152 (24.6%)**	**889 (34.9%)**
Head, face and neck	0	20 (20.6%)	140 (21.9%)	32 (21.1%)	192 (21.6%)
Abdomen	0	21 (21.6%)	150 (23.4%)	44 (28.9%)	215 (24.2%)
Thorax	0	11 (11.3%)	76 (11.9%)	23 (15.1%)	110 (12.4%)
Pelvic	0	20 (20.6%)	77 (12.0%)	20 (13.2%)	117 (13.2%)
Axilla and arm	0	1 (1.0%)	1 (0.2%)	1 (0.7%)	3 (0.3%)
Inguinal region or leg	0	0 (0.0%)	7 (1.1%)	1 (0.7%)	8 (0.9%)
CNS	0	1 (1.0%)	9 (1.4%)	0 (0.0%)	10 (1.1%)
Bone marrow	0	9 (9.3%)	152 (23.8%)	31 (20.4%)	192 (21.6%)
Other	0	14 (14.4%)	28 (4.4%)	0 (0.0%)	42 (4.7%)
Unknown	**0**	**11**	**7**	**4**	**22**

*BL* Burkitt lymphoma, *CNS* central nervous system, *IQR* interquartile range, *NOS* not otherwise specified.

^a^The missing category is not included in the calculation of percentages.

Bold values represent the totals for the subcategories listed in the subsequent rows.

**Fig. 2:**
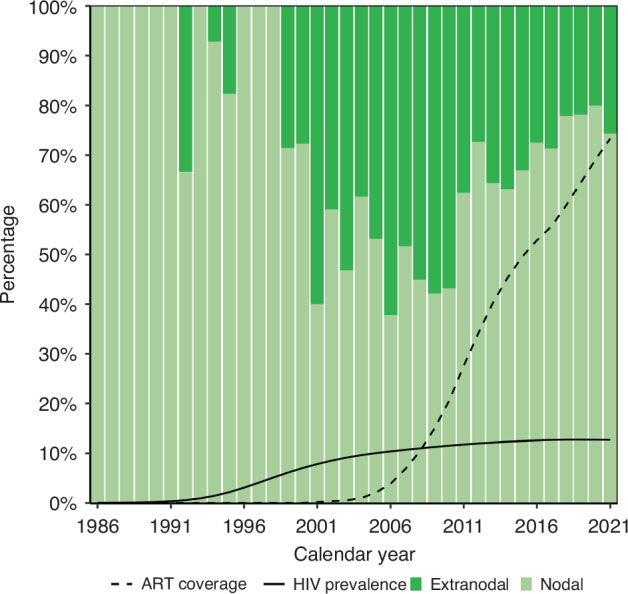
Percentage of nodal and extranodal Burkitt lymphoma over calendar years. ART anti-retroviral treatment, HIV human immunodeficiency virus.

Most BL diagnoses were in lymph nodes (*n* = 1660; 65.1%) but the lymph node location was unspecified in 54.5% (*n* = 905). Until the late 1990s, extranodal BL was uncommon in South Africa, but the proportion of extranodal BL increased rapidly thereafter. Since 2011, the proportion of extranodal BL has decreased again (Fig. 2). All 94 BL diagnosed between 1986–1991 were recorded as nodal, with no further details on location. Among 755 nodal BL with detailed information, 34.8% (*n* = 263) were in the head, neck, and face area; 28.7% (*n* = 217) were in the axilla or arm. Extranodal BL were mostly abdominal (*n* = 215; 24.2%), in the head, neck, and face area (*n* = 192; 21.6%), and in bone marrow (*n* = 192; 21.6%).

**Fig. 3:**
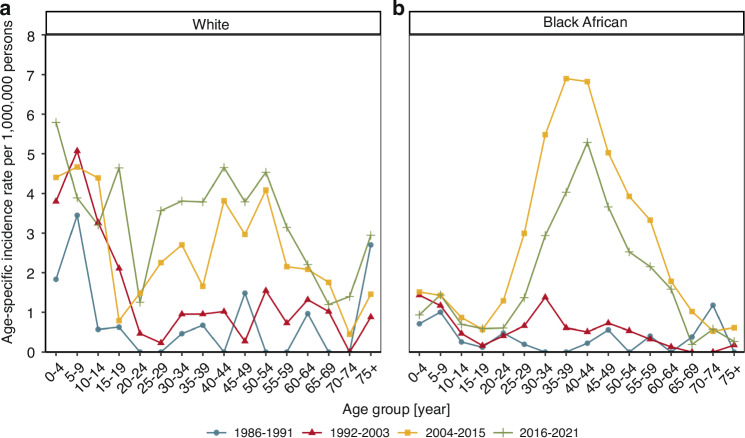
Age (group) specific incidence rate of Burkitt lymphoma by calendar period and race. **a** White population; **b** Black African population.

Over time, the incidence of BL increased across all age groups among both Black African and White individuals (Fig. 3). The White population experienced a consistent rise in BL incidence across most age groups from 1986–2015, followed by a stabilisation during the 2016–2021 period with consistent peaks among children, middle-aged individuals, and the elderly (Fig. 3a). In contrast, the trends in BL incidence among Black Africans were more dynamic. Between 1986–1991, BL rates among Black Africans were generally low across all age groups, with the highest rates observed in children aged 5–9 years and adults aged 70–74 years (Fig. 3b and Supplementary Table 1). However, between 1992–2003, an increase in BL incidence among middle-aged individuals was observable and became much more apparent in the 2004–2015 period. A notable decline in BL incidence rates among Black Africans occurred between 2016–2021, particularly among middle-aged individuals. While the White population generally had higher BL incidence rates across most age groups over the calendar periods, the Black African population had a higher BL incidence among individuals aged 30–49 years during the 2004–2015 period.

The overall ASIR of BL between 1986–2021 was 1.72 per 1,000,000 persons (95% confidence interval [CI] = 1.68–1.75). The ASIR was 1.68 per 1,000,000 Black African (95% CI = 1.63–1.73) and 2.34 per 1,000,000 White persons (95% CI = 2.15–2.53). Among Black Africans, BL risk was higher among male than female individuals overall (IRR = 1.21; 95% CI = 1.11–1.32) but the IRR decreased from 2.42 (95% CI = 1.30–4.51) in 1986–1991 to 1.35 (95% CI = 1.03–1.78) in 1992–2003. In 2004–2015, BL risk was similar in male and female individuals and slightly higher again in male individuals thereafter (Table 2). In contrast, in the White population, the BL risk was at least two-folds higher in male than female individuals throughout all calendar periods.

**Table 2:** Age-standardised incidence rates and male-to-female incidence rate ratio by calendar period.

Race	Calendar period	Overall ASIR (95% CI)	Female ASIR (95% CI)	Male ASIR (95% CI)	Male-to-female IRR (95% CI)
Black African	1986–1991	0.37 (0.28–0.49)	0.22 (0.13–0.35)	0.53 (0.37–0.74)	2.42 (1.30–4.51)
	1992–2003	0.67 (0.59–0.76)	0.57 (0.47–0.68)	0.77 (0.65–0.90)	1.35 (1.03–1.78)
	2004–2015	2.70 (2.56–2.86)	2.58 (2.39–2.79)	2.88 (2.65–3.12)	1.11 (0.98–1.26)
	2016–2021	1.78 (1.63–1.95)	1.61 (1.41–1.82)	1.98 (1.74–2.24)	1.23 (1.01–1.50)
White	1986–1991	0.92 (0.57–1.40)	0.55 (0.21–1.14)	1.30 (0.72–2.14)	2.37 (0.85–6.58)
	1992–2003	1.90 (1.50–2.37)	1.13 (0.72–1.68)	2.65 (1.99–3.45)	2.35 (1.37–4.01)
	2004–2015	2.89 (2.41–3.44)	1.72 (1.24–2.32)	4.07 (3.25–5.02)	2.37 (1.57–3.57)
	2016–2021	3.70 (2.90–4.64)	2.27 (1.44–3.37)	5.15 (3.83–6.74)	2.27 (1.32–3.91)

*ASIR* age-standardised incidence rates, *CI* confidence interval, *IRR* incidence rate ratio.

ASIR per 1,000,000 persons are shown.

**Fig. 4:**
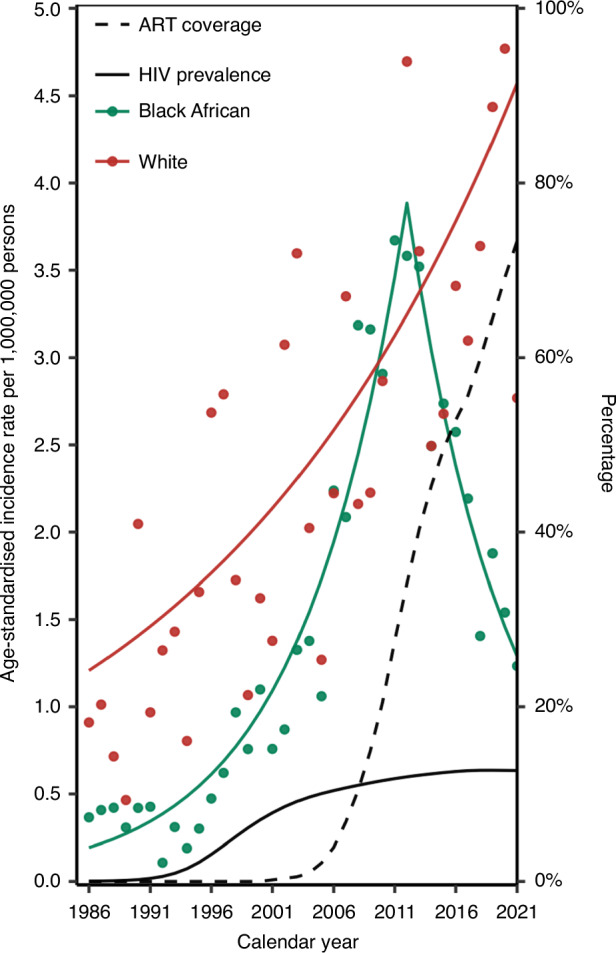
Age-standardised incidence rate (ASIR) of Burkitt lymphoma over calendar years by race. ART anti-retroviral treatment, HIV human immunodeficiency virus.

Between 1986–2012, the ASIR among Black Africans increased yearly by 12.2% (95% CI = 10.4% to 14.1%) and decreased yearly by 11.5% (95% CI = −15.0% to −7.9%) thereafter. Among White individuals, a yearly ASIR increase of 3.9% (95% CI = 2.6% to 5.2%) was noted during the whole study period (Fig. 4).

## Discussion

Between 1986–2021, the temporal trends in the ASIR of BL differed between Black African and White individuals in South Africa. While the ASIR of BL among Black Africans increased from 1986 until 2012 and declined thereafter, it constantly increased among the White population. Over time, the burden of incident BL among Black Africans shifted from children and elderly men to young adults of both sexes, whereas in the White population it increased consistently across all age groups. Extranodal BL, which was previously rare in South Africa, started occurring more frequently over time. Lymph nodes of the head, face and neck area were the most common BL sites across all calendar periods.

This is one of the first country-wide studies in sub-Saharan Africa to characterise the temporal trends of BL incidence rates across all age groups. We examined BL incidence rates over more than three decades of the evolving HIV epidemic using pathologically confirmed diagnoses and comparing two population groups with markedly different HIV prevalence. Our study has some limitations. Not all private laboratories reported to the NCR before cancer reporting became mandatory in 2011. Thus, we may have underestimated the number of incident BL between 1986–2010, particularly among White individuals who frequently access health care in the private sector [26]. Nonetheless, our findings among Black Africans are likely to be representative for the Black African population in South Africa. More than 89% of Black Africans did not have medical insurance by 2010 and accessed care through public-sector health facilities [26], which consistently reported cancer diagnoses to the NCR since 1986. The World Health Organisation (WHO) Classification of BL changed over time, but we used the diagnoses recorded in the NCR and did not reassess records or specimens to standardise classifications. We could not quantify the impact of HIV and EBV on the BL trends at the individual level as HIV-induced immunodeficiency or EBV status were not documented in the NCR database. Including data collected during the COVID-19 pandemic may have affected the temporal trends we observed because health care services were limited during the pandemic and underdiagnosis of BL may have occurred more frequently in 2020–2021.

We found that the age-specific incidence pattern of BL in South Africa changed over time. While the BL incidence rates increased steadily over time in the White population, the changes in the Black African population were dynamic. In 1986–1991, BL incidence among Black Africans followed a bimodal distribution including one peak among children and another peak in elderly people. This pattern is typical for populations with low HIV prevalence [5, 27]. However, from 1992–2003, a BL incidence rate peak emerged among middle-aged individuals, and by 2004–2015, this became the age group predominantly affected by incident BL. The changes in the age-specific pattern of BL rates among Black Africans are likely to be explained by South Africa’s evolving HIV epidemic. The first HIV diagnoses in South Africa were reported in 1982 among men who have sex with men. Subsequent HIV infections mostly occurred among men who have sex with men, blood transfusion recipients, and individuals with haemophilia [28, 29]. HIV was considered rare in South Africa’s general population until 1988 when new infections emerged among heterosexual adults. Subsequently, South Africa experienced a rapid increase in HIV prevalence between 1990 and the early 2000's and a steady increase thereafter [28]. Since 2015, the HIV prevalence has stabilised at around 13% in the general population [23]. Black Africans are most heavily affected by HIV with an HIV prevalence of 16.6% compared to 1.1% among White individuals in 2017 [15]. The HIV prevalence in South Africa is highest among individuals aged 15–49 years [23]. As young and middle-aged individuals became predominantly affected by HIV, their BL risk also increased [8, 9]. Similar changes in the age distribution of incident BL have been described in other countries. In the US, where the HIV prevalence is higher among male than female individuals, the age-specific BL rates peak at ages 10, 40 and over 75 years among male individuals and at ages 10 and over 75 years among female individuals [5, 18]. An emerging BL incidence peak among middle-aged men was also observed in Europe, Asia, Central and South America at the onset of the HIV epidemic in the 1980’s [27], whereas in South Africa a similar peak started appearing in 2004. This lag may be partly explained by BL initially being misdiagnosed as tuberculosis as the two diseases share common symptoms [30] and the high HIV mortality before the introduction of ART at public health facilities in 2004 [5]. The steady increase in BL incidence over calendar periods across age groups in the White population may reflect the low HIV prevalence in this group.

Male individuals have a two- to four-fold higher BL risk than female individuals in most geographic regions [4]. The increased BL risk in male individuals is not well understood, but factors such as hormonal and immunological differences, as well as differences in carcinogen exposure might play a role [4, 31–33]. We found that the male-to-female IRR of BL among the White population remained constantly above two, whereas it decreased from 2.42 in 1986–1991 to 1.11 in 2004–2015 and 1.23 in 2016–2021 among Black Africans. The temporal changes in the male-to female IRR among Black Africans with a more equal BL risk distribution between the sexes in recent years are likely to be driven by the HIV epidemic. Since the 1990s, the HIV prevalence has been higher among female than male individuals in South Africa [23]. In 2021, approximately 65% of South Africa’s PWH aged 15–49 years were female [23]. In the US, on the other hand, HIV predominantly affects men who already have a higher BL risk and thus, a US-based study found consistently higher BL rates among men than women across all calendar periods [5].

Studies have demonstrated a decreasing incidence of HIV-associated cancers with increasing ART coverage. For instance, in many parts of the world a decline in the incidence of Kaposi sarcoma and non-Hodgkin lymphoma was reported with the introduction of ART [34, 35]. In South Africa, Kaposi sarcoma rates in the Black African population rose with increasing HIV prevalence but started declining in 2008 [36]. Conjunctival squamous cell carcinoma, another HIV-associated malignancy, followed a similar pattern in South Africa’s general population with the decline starting in 2009 [37]. Likewise, among Black Africans, we observed increasing BL incidence rates until 2012 followed by a declining trend starting 9 years after ART introduction. Similarly, in the US, a declining BL incidence trend across all population groups only became evident from 2009, despite ART being available from 1996 [18, 38]. Thus, the recent decline in the BL incidence rate among Black Africans could be partly explained by the increasing ART coverage over time. The reasons for the steady increase in BL incidence rates over time among White individuals in South Africa are not well understood. However, it has been hypothesised that advancement in medical technology and diagnostic capabilities may have contributed to a global increase in the incidence of BL between 1990–2021 [39]. Other factors such as temporal changes in the BL classification guidelines may also have affected the BL incidence trends we observed. A significant change in the WHO classification of tumours of haematopoietic and lymphoid tissues occurred in 2008, when Burkitt-like lymphoma was no longer classified as BL [40–42], and this change has been associated with a subsequent decline in recorded BL incidence in the US. Challenges in distinguishing BL from other aggressive non-Hodgkin lymphomas and resulting misclassifications may also have contributed to the observed BL rates over time, especially as the classifications keep evolving and become more complex.

Although African countries bear the highest burden of HIV in the world, very few studies in the region have examined BL incidence rates among adults [3]. Additional investigations on the effect of immunodeficiency, virological control and ART on incident BL and continuous monitoring of temporal trends are crucial to inform public health strategies, resource allocation, and the development of targeted interventions to address the changing landscape of BL incidence in South Africa. Meanwhile, clinicians in a high HIV prevalence setting like South Africa should be aware that BL most commonly occurs in middle-aged men and women and consider this when evaluating individuals that present with enlarged lymph nodes or intra-abdominal masses, especially among PWH.

In conclusion, we found that BL incidence trends and demographics in South Africa changed over time as the HIV epidemic evolved, with BL rates among Black Africans starting to decline in 2012. In contrast, BL rates continuously increased among White individuals, in line with studies from high-income countries reporting increasing or stabilising BL rates over time. Further research is needed to better understand the differences in BL epidemiology across geographic regions and population groups.

## Supplementary information

Supplemental material

## Data Availability

National Cancer Registry data supporting the findings of this study are available upon reasonable request from the South African NCR through the National Health Laboratory Service Academic Affairs and Research Management System (https://aarms.nhls.ac.za). Population statistics were obtained from published Statistics South Africa reports (https://www.statssa.gov.za/?page_id=1854&PPN=P0302). HIV and ART data were derived from published Thembisa Project reports (https://thembisa.org/downloads).

## Code availability

The code used for data analysis and figure generation in this study is available upon request. Interested researchers can contact the corresponding author to obtain the scripts and relevant documentation.
