# Supplementary material for: Patterns of incident Burkitt lymphoma during the HIV epidemic among the Black African and White population in South Africa
Source: Br J Cancer. 2025 Jan 14;132(5):462–8. doi: 10.1038/s41416-024-02937-8 (PMC11876306; doi:10.1038/s41416-024-02937-8)
Supplement: Supplementary file 1 — Supplemental material [file 41416_2024_2937_MOESM1_ESM.docx]

SUPPLEMENTARY MATERIAL

Patterns of incident Burkitt lymphoma during the HIV epidemic among the Black African and White population in South Africa

Supplementary Table 1: Age-specific incidence rate by sex and calendar period per 1,000,000 persons

| **Age-group  [years]** | **Overall** | | | | **Black African** | | | | **White** | | | |
| --- | --- | --- | --- | --- | --- | --- | --- | --- | --- | --- | --- | --- |
|  | ***1986-1991  ASR (95% CI)*** | ***1992-2003  ASR (95% CI)*** | ***2004-2015  ASR (95% CI)*** | ***2016-2021  ASR (95% CI)*** | ***1986-1991  ASR (95% CI)*** | ***1992-2003  ASR (95% CI)*** | ***2004-2015  ASR (95% CI)*** | ***2016-2021  ASR (95% CI)*** | ***1986-1991  ASR (95% CI)*** | ***1992-2003  ASR (95% CI)*** | ***2004-2015  ASR (95% CI)*** | ***2016-2021  ASR (95% CI)*** |
| 0-4 | 0.8 (0.6 - 1.1) | 1.6 (1.4 - 1.8) | 1.7 (1.5 - 1.9) | 1.2 (0.9 - 1.4) | 0.7 (0.4 - 1.2) | 1.4 (1.1 - 1.8) | 1.5 (1.2 - 1.9) | 0.9 (0.6 - 1.4) | 1.8 (0.4 - 5.0) | 3.8 (2.0 - 6.5) | 4.4 (2.4 - 7.4) | 5.8 (2.5 - 11.4) |
| 5-9 | 1.2 (1.0 - 1.5) | 1.5 (1.3 - 1.7) | 1.6 (1.4 - 1.8) | 1.6 (1.3 - 1.8) | 1.0 (0.6 - 1.5) | 1.2 (0.9 - 1.5) | 1.4 (1.1 - 1.8) | 1.4 (1.0 - 1.9) | 3.5 (1.4 - 6.9) | 5.1 (3.1 - 7.9) | 4.7 (2.6 - 7.7) | 3.9 (1.4 - 8.5) |
| 10-14 | 0.3 (0.2 - 0.5) | 0.7 (0.6 - 0.8) | 1.1 (0.9 - 1.3) | 0.8 (0.7 - 1.0) | 0.3 (0.1 - 0.6) | 0.5 (0.3 - 0.7) | 0.9 (0.6 - 1.2) | 0.7 (0.4 - 1.1) | 0.6 (0.0 - 2.4) | 3.3 (1.8 - 5.5) | 4.4 (2.5 - 7.2) | 3.2 (1.0 - 7.5) |
| 15-19 | 0.2 (0.1 - 0.3) | 0.3 (0.3 - 0.5) | 0.6 (0.5 - 0.7) | 0.8 (0.6 - 1.1) | 0.1 (0.0 - 0.4) | 0.2 (0.1 - 0.3) | 0.6 (0.4 - 0.8) | 0.6 (0.3 - 1.0) | 0.6 (0.0 - 2.7) | 2.1 (1.0 - 4.0) | 0.8 (0.2 - 2.3) | 4.6 (1.9 - 9.6) |
| 20-24 | 0.4 (0.3 - 0.6) | 0.4 (0.3 - 0.5) | 1.3 (1.1 - 1.5) | 0.6 (0.5 - 0.8) | 0.5 (0.2 - 1.0) | 0.4 (0.2 - 0.7) | 1.3 (1.0 - 1.6) | 0.6 (0.3 - 1.0) | 0.0 (0.0 - 1.6) | 0.5 (0.1 - 1.7) | 1.5 (0.5 - 3.2) | 1.3 (0.2 - 4.5) |
| 25-29 | 0.2 (0.1 - 0.3) | 0.6 (0.5 - 0.8) | 2.9 (2.7 - 3.2) | 1.5 (1.3 - 1.8) | 0.2 (0.0 - 0.6) | 0.7 (0.4 - 1.0) | 3.0 (2.5 - 3.5) | 1.4 (1.0 - 1.9) | 0.0 (0.0 - 1.6) | 0.2 (0.0 - 1.3) | 2.3 (1.0 - 4.3) | 3.6 (1.3 - 7.8) |
| 30-34 | 0.1 (0.0 - 0.2) | 1.3 (1.1 - 1.6) | 5.2 (4.8 - 5.6) | 3.0 (2.6 - 3.4) | 0.0 (0.0 - 0.3) | 1.4 (1.0 - 1.9) | 5.5 (4.8 - 6.3) | 2.9 (2.3 - 3.7) | 0.5 (0.0 - 2.6) | 1.0 (0.3 - 2.4) | 2.7 (1.3 - 4.8) | 3.8 (1.5 - 7.8) |
| 35-39 | 0.1 (0.0 - 0.3) | 0.7 (0.5 - 0.9) | 6.3 (5.8 - 6.8) | 4.0 (3.6 - 4.5) | 0.0 (0.0 - 0.4) | 0.6 (0.3 - 1.0) | 6.9 (6.0 - 7.9) | 4.0 (3.2 - 5.0) | 0.7 (0.0 - 2.9) | 1.0 (0.3 - 2.4) | 1.7 (0.7 - 3.4) | 3.8 (1.5 - 7.8) |
| 40-44 | 0.2 (0.1 - 0.4) | 0.6 (0.4 - 0.8) | 6.4 (5.9 - 6.9) | 5.2 (4.6 - 5.8) | 0.2 (0.0 - 1.0) | 0.5 (0.2 - 0.9) | 6.8 (5.9 - 7.9) | 5.3 (4.3 - 6.5) | 0.0 (0.0 - 1.9) | 1.0 (0.3 - 2.6) | 3.8 (2.2 - 6.2) | 4.7 (2.1 - 8.8) |
| 45-49 | 0.8 (0.4 - 1.3) | 0.6 (0.4 - 0.9) | 4.7 (4.2 - 5.2) | 3.7 (3.1 - 4.3) | 0.6 (0.1 - 1.6) | 0.7 (0.4 - 1.3) | 5.0 (4.1 - 6.1) | 3.7 (2.7 - 4.8) | 1.5 (0.2 - 4.8) | 0.3 (0.0 - 1.5) | 3.0 (1.5 - 5.2) | 3.8 (1.6 - 7.5) |
| 50-54 | 0.0 (0.0 - 0.2) | 0.8 (0.5 - 1.1) | 4.0 (3.5 - 4.5) | 2.8 (2.3 - 3.4) | 0.0 (0.0 - 0.9) | 0.5 (0.2 - 1.2) | 3.9 (3.1 - 5.0) | 2.5 (1.7 - 3.7) | 0.0 (0.0 - 2.7) | 1.5 (0.5 - 3.6) | 4.1 (2.3 - 6.6) | 4.5 (2.1 - 8.6) |
| 55-59 | 0.3 (0.1 - 0.7) | 0.4 (0.2 - 0.7) | 3.1 (2.6 - 3.6) | 2.3 (1.8 - 2.9) | 0.4 (0.0 - 1.7) | 0.3 (0.1 - 0.9) | 3.3 (2.4 - 4.4) | 2.2 (1.3 - 3.4) | 0.0 (0.0 - 3.1) | 0.7 (0.1 - 2.6) | 2.2 (0.9 - 4.2) | 3.1 (1.2 - 6.8) |
| 60-64 | 0.2 (0.1 - 0.7) | 0.4 (0.2 - 0.7) | 1.9 (1.5 - 2.3) | 1.7 (1.2 - 2.3) | 0.0 (0.0 - 1.2) | 0.1 (0.0 - 0.7) | 1.8 (1.1 - 2.8) | 1.6 (0.8 - 2.8) | 1.0 (0.0 - 5.4) | 1.3 (0.3 - 3.8) | 2.1 (0.8 - 4.3) | 2.2 (0.6 - 5.6) |
| 65-69 | 0.3 (0.1 - 0.8) | 0.2 (0.1 - 0.5) | 1.2 (0.9 - 1.7) | 0.4 (0.2 - 0.8) | 0.4 (0.0 - 2.1) | 0.0 (0.0 - 0.6) | 1.0 (0.4 - 2.0) | 0.2 (0.0 - 1.1) | 0.0 (0.0 - 4.1) | 1.0 (0.1 - 3.7) | 1.8 (0.6 - 4.1) | 1.2 (0.1 - 4.3) |
| 70-74 | 0.7 (0.2 - 1.7) | 0.0 (0.0 - 0.2) | 0.5 (0.3 - 0.9) | 0.8 (0.4 - 1.4) | 1.2 (0.1 - 5.0) | 0.0 (0.0 - 0.9) | 0.5 (0.1 - 1.5) | 0.6 (0.1 - 2.1) | 0.0 (0.0 - 4.3) | 0.0 (0.0 - 2.4) | 0.5 (0.0 - 2.5) | 1.4 (0.2 - 5.0) |
| 75+ | 1.0 (0.4 - 1.8) | 0.4 (0.2 - 0.7) | 0.9 (0.6 - 1.3) | 1.3 (0.8 - 2.0) | 0.0 (0.0 - 1.8) | 0.2 (0.0 - 1.0) | 0.6 (0.2 - 1.6) | 0.3 (0.0 - 1.5) | 2.7 (0.6 - 7.9) | 0.9 (0.1 - 3.2) | 1.5 (0.5 - 3.4) | 2.9 (1.2 - 6.1) |

ASR: Age-specific rates; CI: Confidence Interval
